# Supplementary material for: Supervised Learning in Spiking Neural Networks for Precise Temporal Encoding
Source: PLoS One. 2016 Aug 17;11(8):e0161335. doi: 10.1371/journal.pone.0161335 (PMC4988787; doi:10.1371/journal.pone.0161335)
Supplement: S1 Text — (PDF) [file pone.0161335.s001.pdf]

Supervised learning in spiking neural networks for precise temporal encoding

S1 Text: Convergence of gradient ascent procedure based on intrinsic neuronal dynamics

Brian Gardner*, André Grüning
Department of Computer Science, University of Surrey, Guildford, U.K.
*** E-mail: b.gardner@surrey.ac.uk**

In our approach we wish to consider a learning rule that depends on the intrinsic dynamics of a postsynaptic neuron, such that synaptic weights are modified based on actual generated output spikes. This contrasts with the theoretically derived, gradient ascent learning rule of Eq. (12, main text), which is restated below for convenience:

$$\Delta w_{ij}^{\mathrm{ref}} = \frac{\eta}{\Delta u} \int_0^T \left[\mathcal{Y}_i^{\mathrm{ref}}(t) - \rho_i(t|\mathbf{x}, y_i^{\mathrm{ref}}) \right] \sum_{t_j^f \in x_j} \epsilon(t - t_j^f) \, \mathrm{d}t \ , \tag{S1}$$

where the stochastic intensity $\rho_i(t|\mathbf{x}, y_i^{\mathrm{ref}})$ signifies that the neuron's firing activity has been artificially clamped to its target response. We instead desire synaptic weight modifications informed by the neuron's intrinsic firing activity, as given by Eq. (13, main text), which is also restated below:

$$\Delta w_{ij} = \frac{\eta}{\Delta u} \int_0^T \left[\mathcal{Y}_i^{\mathrm{ref}}(t) - \rho_i(t|\mathbf{x}, y_i) \right] \sum_{t_j^f \in x_j} \epsilon(t - t_j^f) \, \mathrm{d}t \ , \tag{S2}$$

where we have substituted $\rho_i(t|\mathbf{x}, y_i^{\mathrm{ref}})$ with $\rho_i(t|\mathbf{x}, y_i)$, such that the stochastic intensity of the postsynaptic neuron depends on its actual sequence of emitted output spikes y_i rather than its target output y_i^{ref}. In this supplementary text, we show that Eq. (S2) yields similar weight updates to Eq. (S1) if the actual postsynaptic spike train is already close to its target.

To demonstrate this, we start by considering the absolute difference between weight updates applied using Eqs. (S2) and (S1):

$$\left| \Delta w_{ij} - \Delta w_{ij}^{\mathrm{ref}} \right| = \frac{\eta}{\Delta u} \left| \int_0^T \left[\rho_i(t|\mathbf{x}, y_i) - \rho_i(t|\mathbf{x}, y_i^{\mathrm{ref}}) \right] \sum_{t_j^f \in x_j} \epsilon(t - t_j^f) \, \mathrm{d}t \right| \ , \tag{S3}$$

leading to the following inequality for an absolute integrand:

$$|\Delta w_{ij} - \Delta w_{ij}^{\mathrm{ref}}| \leq \frac{\eta}{\Delta u} \int_0^T \left| \rho_i(t|\mathbf{x}, y_i) - \rho_i(t|\mathbf{x}, y_i^{\mathrm{ref}}) \right| \sum_{t_j^f \in x_j} \epsilon(t - t_j^f) \, \mathrm{d}t \ . \tag{S4}$$

Now, for simplicity, if we assume one of the presynaptic neurons, denoted j, contributes a single input spike at time $t_j = 0\,\mathrm{ms}$, and a single target and actual output spike occur at times t_i^{ref} and t_i for a postsynaptic neuron i, respectively, then the above equation simplifies to

$$|\Delta w_{ij} - \Delta w_{ij}^{\mathrm{ref}}| \leq \frac{\eta}{\Delta u} \int_0^T \left|\rho_i(t|\mathbf{x}, t_i) - \rho_i(t|\mathbf{x}, t_i^{\mathrm{ref}})\right| \epsilon(t)\, \mathrm{d}t \; . \tag{S5}$$

Here, $\rho_i(t|\mathbf{x}, t_i)$ denotes a dependence of the postsynaptic neuron's stochastic intensity at time t on the entire set of presynaptic spikes $\mathbf{x}$, including from neuron j, and its actual output firing time t_i.

According to the definition of the PSP kernel in Eq. (4, main text) the kernel assumes a maximum value, denoted ϵ^{peak}. Hence, an upper bound of Eq. (S5) can be given by

$$|\Delta w_{ij} - \Delta w_{ij}^{\mathrm{ref}}| \leq \frac{\eta\, \epsilon^{\mathrm{peak}}}{\Delta u} \int_0^T \left|\rho_i(t|\mathbf{x}, t_i) - \rho_i(t|\mathbf{x}, t_i^{\mathrm{ref}})\right| \, \mathrm{d}t \; . \tag{S6}$$

We emphasise here that although we consider just a single input spike, the above would equally be valid for multiple input spikes by simply multiplying the upper bound on the PSP, ϵ^{peak}, by the number of spikes contributed from neuron j.

As defined by Eq. (7, main text), the stochastic intensity has an exponential dependence on the postsynaptic neuron's membrane potential, and the only difference between ρ_i and ρ_i^{ref} arises from their reset term κ, hence Eq. (S6) can be written as

$$|\Delta w_{ij} - \Delta w_{ij}^{\mathrm{ref}}| \leq \frac{\eta\, \epsilon^{\mathrm{peak}}}{\Delta u} \int_0^T \rho_i(t|\mathbf{x}, t_i^{\mathrm{ref}}) \left|\exp\left(\frac{\kappa(t - t_i) - \kappa(t - t_i^{\mathrm{ref}})}{\Delta u}\right) - 1\right| \mathrm{d}t \; . \tag{S7}$$

For a given finite set of inputs (all predecessor neurons of i) on the finite interval $[0, T]$, u_i is smaller than a constant, irrespective of where the target and actual output spikes fall (consider the maximum of u_i in Eq. (1, main text) for y_i the empty set). Therefore, ρ_i is also bounded by a constant ρ_i^{max}, and hence

$$|\Delta w_{ij} - \Delta w_{ij}^{\mathrm{ref}}| \leq \frac{\eta\, \epsilon^{\mathrm{peak}}\, \rho_i^{\mathrm{max}}}{\Delta u} \int_0^T \left|\exp\left(\frac{\kappa(t - t_i) - \kappa(t - t_i^{\mathrm{ref}})}{\Delta u}\right) - 1\right| \mathrm{d}t \; . \tag{S8}$$

We now show in the following that the integral on the right-hand side of the above equation, and by extension the difference in the weight change, becomes small if t_i and t_i^{ref} are close together. If we assume $\delta t := t_i - t_i^{\mathrm{ref}} > 0$ (an analogous argument applies also for $\delta t < 0$), then the difference between the reset kernels can be expressed as

$$K(t) := \kappa(t - t_i) - \kappa(t - t_i^{\mathrm{ref}}) \; , \tag{S9}$$

and the absolute difference

$$\begin{aligned} |K(t)| &= |\kappa_0| \left|\exp\left(-\frac{t - t_i}{\tau_m}\right) \Theta(t - t_i) - \exp\left(-\frac{t - t_i^{\mathrm{ref}}}{\tau_m}\right) \Theta(t - t_i^{\mathrm{ref}})\right| \\ &\leq |\kappa_0| \left|\exp\left(-\frac{t - t_i}{\tau_m}\right) - \exp\left(-\frac{t - t_i^{\mathrm{ref}}}{\tau_m}\right)\right| \Theta(t - t_i) \\ &\quad + |\kappa_0| \exp\left(-\frac{t - t_i^{\mathrm{ref}}}{\tau_m}\right) \mathbb{1}_A(t) \; , \end{aligned} \tag{S10}$$

where $\mathbb{1}_A(t)$ is the indicator function on the interval $A = [t_i^{\text{ref}}, t_i]$, such that $\mathbb{1}_A(t) = 1$ if $t \in A$, and $\mathbb{1}_A(t) = 0$ otherwise. Furthermore, the following inequality applies:

$$|K(t)| \leq |\kappa_0| \exp\left(-\frac{t - t_i}{\tau_m}\right) \left|1 - \exp\left(-\frac{\delta t}{\tau_m}\right)\right| \Theta(t - t_i) + |\kappa_0| \mathbb{1}_A(t) \ . \qquad \text{(S11)}$$

Hence, $|K(t)|$ tends to zero point-wise in t for $\delta t \to 0$ and is bounded by $|\kappa_0|$. By continuity, the integrand in Eq. (S8), $|\exp(K(t)) - 1|$, also goes to zero pointwise and is bounded. Hence by dominated convergence, the integral in (S8), tends to zero for $\delta t \to 0$. In other words, it is continuous in δt, and we can find for each ε a δt such that weight changes based on t_i do not differ by more than ε from those based on t_i^{ref} if target and actual output spikes are closer than δt.

The proof above shows that the intrinsic weight update rule of Eq. (S2) yields comparable weight changes to the rule in Eq. (S1) if the actual output spike is already close to its target. However, in the form given above it is not constructive, i.e. does not give us an explicit estimate of δt in terms of ε and other parameters. As for all learning rules, their practical feasibility has to be demonstrated in simulations.
